# What do medical students actually need to know about artificial intelligence?

**DOI:** 10.1038/s41746-020-0294-7

**Published:** 2020-06-19

**Authors:** Liam G. McCoy, Sujay Nagaraj, Felipe Morgado, Vinyas Harish, Sunit Das, Leo Anthony Celi

**Affiliations:** 1 Faculty of Medicine, University of Toronto, Medical Sciences Building, 1 King’s College Cir, Toronto, ON M5S 1A8 Canada; 2 Institute of Health Policy, Management and Evaluation, Dalla Lana School of Public Health, University of Toronto, 155 College St 4th Floor, Toronto, ON M5T 3M6 Canada; 3 Department of Computer Science, University of Toronto, 40 St. George Street, Room 4283, Toronto, ON M5S 2E4 Canada; 4 Department of Medical Biophysics, University of Toronto, 101 College St, Suite 15-701, Toronto, ON M5G 1L7 Canada; 5 Centre for Ethics, University of Toronto, 15 Devonshire Pl, Toronto, ON M5S 1H8 Canada; 6 Institute for Medical Engineering and Science, Massachusetts Institute of Technology, 77 Massachusetts Avenue, E25-505, Cambridge, MA 02139 USA; 7 Division of Pulmonary, Critical Care and Sleep Medicine, Beth Israel Deaconess Medical Center, 330 Brookline Avenue, Boston, MA 02215 USA; 8 Department of Biostatistics, Harvard T.H. Chan School of Public Health, 677 Huntington Avenue, Boston, MA 02115 USA

**Keywords:** Health care, Medical ethics, Health care, Medical ethics

## Abstract

With emerging innovations in artificial intelligence (AI) poised to substantially impact medical practice, interest in training current and future physicians about the technology is growing. Alongside comes the question of what, precisely, should medical students be taught. While competencies for the clinical usage of AI are broadly similar to those for any other novel technology, there are qualitative differences of critical importance to concerns regarding explainability, health equity, and data security. Drawing on experiences at the University of Toronto Faculty of Medicine and MIT Critical Data’s “datathons”, the authors advocate for a dual-focused approach: combining robust data science-focused additions to baseline health research curricula and extracurricular programs to cultivate leadership in this space.

## Introduction

With emerging innovations in artificial intelligence (AI) poised to substantially impact medical practice, interest in training current and future physicians about AI is growing^1^. Alongside this interest comes the question of what, precisely, medical students should learn^2^. While competencies for the clinical usage of AI are broadly similar to those for any other novel technology in medicine, there are qualitative differences of critical importance to concerns regarding explainability, health equity, and data security^3–5^. We advocate for a dual-focused approach: combining robust, learner-centered AI additions to baseline curricula and extracurricular programs to cultivate leadership in this space.

## What do physicians need to understand about AI in the clinical context?

Most directly, physicians need to understand AI in the same way that they need to understand any technology impacting clinical decision-making. A physician utilizing MRI, for example, does not need to understand the particle spin physics differentiating T1 and T2 weighted scans, but they do need to be able to:

(i) Use it—identify when the technology is appropriate for a given clinical context, and what inputs are required to receive meaningful results.
(ii) Interpret it—understand and interpret the results with a reasonable degree of accuracy, including awareness of sources of error, bias, or clinical inapplicability.
(iii) Explain it—be able to communicate the results and the processes underlying them in a way that others (e.g. allied health professionals and patients) can understand.

These skills take on particular nuances in the context of AI. For (i) and (ii), it is critical for physicians to appreciate the highly context-specific nature of AI, and the fact that performance in a single restricted context may not always be transferable. It is also important to be aware of factors which may decrease the performance of algorithms for specific patient groups^3^.

AI has been commonly criticized for the “black box” effect—that is, the mechanism by which a model arrives at a decision may be indecipherable^1^. This lack of technical “explainability”, however, does not discharge the obligations of (iii). To satisfy requirements of informed consent and clinical collaboration, a physician may be called upon to communicate their understanding of the origin, nature, and justification of an algorithm’s results to patients, families, and colleagues.

## What do physicians need to understand about AI in the broader professional context?

The professional obligations of physicians extend beyond the clinical role into leadership and health advocacy. The disruptive prospects of AI in healthcare raise significant ethical and operational challenges which physicians must collectively be prepared to engage with for the sake of ensuring patient welfare.

Substantial concerns exist regarding the impact of algorithmic clinical decision support on health equity, due to factors such as the use of datasets lacking representation from minority populations^3^, and the possibility for algorithms to learn from and perpetuate existing biases^4^. Risks around data security and privacy are also becoming rapidly apparent^5^. There is also, however, the potential for AI itself to alleviate some of medicine’s existing problems with bias and unfairness^6^. Physicians should be aware of both possibilities and be equipped to advocate for the development and deployment of ethical and equitable systems. Finally, physicians must act as responsible stewards for patient data to ensure that the foundational trust between provider and patient is not violated.

## How might medical students learn what they need to learn?

Concerted efforts should be taken to cultivate physician-leaders who are fluent in both AI and medicine. Such dual competence is important, as it is no simple task to select clinically relevant and computationally feasible targets for AI in medicine. A siloed approach may lead to clear clinical targets going unnoticed and worsen the production of technical “solutions in search of problems”^7^. A multidisciplinary, integrated approach to learning will serve to facilitate this goal.

When approaching such a complex topic, it is critical to distinguish between that which all physicians must know for everyday practice, and that which some physicians should know to drive innovation. Curricular components should be targeted to address the former, while robust extracurricular programs can be targeted toward the latter. Both components serve to promote discussions on how the convergence between AI and medicine is currently impacting and will continue to impact the physician’s identity. This aligns with the concept of the “reimagined medical school”, which establishes a framework of core knowledge while supporting students who seek deep dives into specific subject areas^8^.

This approach has been piloted at the University of Toronto (UofT) Faculty of Medicine and has been embraced by administration as an important part of the Faculty’s strategic plan^8^. Lectures in the preclinical curriculum introduce all students to these concepts, and the 2-year-long “Computing for Medicine” certificate program provides particularly interested students with practical programming skills and immersion into clinical data science projects^9^. Additionally, an “AI in Medicine” student interest group hosts extracurricular seminars on the subject and helps to facilitate connections between medical students and a city’s broader AI ecosystem (in academia and industry) (see Supplementary Table 1 for a list of AI in Medicine offerings in the last two years).

Harvard Medical School has engaged in a similar approach, offering clinical informatics training as an elective for medical students^10^. During this elective, students are paired with faculty mentors in their area of interest and engage in a mix of didactic and hands-on learning to explore how informatics is embedded into health systems. The School has also collaborated with the MIT Critical Data group to offer a project-based course on data science in medicine^11^. Extracurricularly, the MIT Critical Data Group has worked to spur interest in AI through “datathons” (brief competitions wherein computer scientists and clinicians work together to use data to solve clinical problems)^12^. These collaborations are emblematic of the possibilities for collaboration with non-medical faculties to enrich the education of medical students.

With insight from these experiences, we identify a series of important opportunities in both the curricular and extracurricular realms (outlined in Table 1). We wish to emphasize the importance of finding synergy between the learning objectives and their delivery, and of maintaining a learner-centered ethos with a focus upon student engagement rather than passive knowledge transfer. These concepts should be integrated with other aspects of the curriculum wherever appropriate (such as the inclusion of an AI case study in a workshop about ethical clinical decision-making), as the competencies required to effectively work with AI will often overlap with those required to fulfil other core aspects of the physician role such as advocacy, leadership, and communication. Medical schools have a critical role to play not only in helping their students learn but also in nurturing their academic interests and sowing the seeds of future leadership. These recommendations can and should be tailored to the context and strengths of each medical school, its partnerships, and its student body.

**Table 1:** Potential curricular and extracurricular learning opportunities for artificial intelligence in medicine.

Curricular objective	Delivery recommendations	Extracurricular objective	Delivery recommendations
Promote physicians to be data-savvy consumers Students should be able to critically evaluate AI claims and understand the connection between models and clinical realities.	Actively engage students with hands-on workshops focused around: Recognizing appropriate potential applications of AI to health data Understanding how to discern between different methods that can be applied to data (e.g. the distinction between prediction and causal inference approaches)	Promote student interest groups in AI Interested students should be encouraged to connect and build networks around their shared AI focus.	Extend the broadly-used format of the “student interest group” to AI, enabling students to organize and autonomously host initiatives such as: Seminar events with prominent AI in Medicine speakers Hackathons and datathons in collaboration with computer science and engineering students
Instill durable fundamental concepts about AI, while avoiding technical specifics It is more important for students to have a robust conceptual understanding of AI and the structure of clinical data science than to understand constantly changing technical specifics.	Incorporate lecture and self-learning module content around: The basic pipeline of data acquisition, cleaning, analysis, and visualization Issues with data stewardship and data quality assurance in healthcare. Classes of machine learning approaches and common issues with design and integration of AI into clinical practice	Facilitate connections between medical students and industry in the health-AI space As AI in medicine is not siloed to solely academia or industry, students should have the opportunity to be exposed to the AI ecosystem in their local and broader communities	Leverage partnerships (either at the Faculty level or the student group level) to offer: Student site visits to start-ups to learn about entrepreneurship and the creation of health AI products and services Student research opportunities with health AI companies, or public-private partnerships
Introduce frameworks for approaching ethical considerations, both clinically and at a systems level Students should appreciate fairness, accountability, and transparency as core AI analogues to the traditional bioethics principles of beneficence, non-maleficence, autonomy, and justice^15^.	Students should participate in interactive case-based workshops and seminars lead by AI and ethics experts focused on: The special considerations AI requires at clinical and system levels in a case-based format How fairness, accountability, and transparency directly relate to core clinical values of beneficence, non-maleficence, autonomy, and justice which must permeate through all aspects of their care	Provide longitudinal programs to give students hands-on experience with real-world AI projects Theoretical knowledge should be supplemented with practical, real-world experience through formalized programs.	Longitudinal programs can include but are not limited to: “Computing for Medicine”, a validated 14-week course offered to preclinical students in UofT’s MD program to promote computer literacy, algorithmic thinking, and cross-domain collaboration^9^ Non-technical projects involving AI or data science, such as using design-thinking approaches to implement existing AI tools into clinical practice and workflows
Promote computer science/data science as a dual-training path for MD/PhD and MD/MSc students Students should be provided with partnered, formalized learning opportunities that provide training at the intersection of health and data science.	Establish partnerships with institutes across computer science, biomedical engineering, the basic sciences, and public health, such as: U of T Faculty of Medicine’s partnerships with the Vector Institute for Artificial Intelligence, and Schwartz Reisman Institute for Technology and Society Harvard Medical School’s Collaborative Health Sciences and Technology MD/PhD Offerings with MIT	Encourage cross-disciplinary collaborations between medical students and data scientists Students should build interdisciplinary networks, and be encouraged to connect and collaborate with peers across faculties	Take active steps to break past disciplinary silos through initiatives such as: Shared “AI in Medicine” journal clubs open to both medical students and computer science/engineering students Collaborative events such as “datathons”, wherein ad-hoc interdisciplinary teams compete to answer clinical questions on open database

## What about after medical school?

While detailed discussion on postgraduate medical education (PGME) and continuing medical education (CME) is outside the scope of this work, it is important to consider that medical education is viewed as a life-long pursuit and attention needs to be provided to learners at later career stages^13^. Competencies around AI could be integrated in PGME curricula in existing research or Quality Improvement (QI) blocks. Research training, for medical or surgical trainees, could be in technical areas such as data science or biomedical engineering but also in ethics, health services research, and medical education. QI would focus on translating and evaluating proven innovations into care. CME offerings through online or in-person workshops can not only allow clinicians to refresh their competencies over the course of their career but also empower established practitioners with the skills and knowledge to keep up with this field^14^. The various curricular aspects in Table 1 can be modified to suit learners at different stages in their careers.

## Conclusion

Ultimately, medical schools are tasked with training physicians for a future in which artificial intelligence is poised to play a significant role. In order to succeed at this task, it will be essential for students to have curricular and extracurricular learning opportunities around the clinical usage, technical limitations, and ethical implications of the tools at their disposal. Given the importance and potential impact of this technology, we must act both to ensure a base of artificial intelligence literacy among physicians at-large and to nurture the skills and interests of the future leaders who will drive innovation in this space.

## Supplementary information

Supplementary Information
