# Supplementary material for: What do medical students actually need to know about artificial intelligence?
Source: NPJ Digit Med. 2020 Jun 19;3:86. doi: 10.1038/s41746-020-0294-7 (PMC7305136; doi:10.1038/s41746-020-0294-7)
Supplement: Supplementary file 1 — Supplementary Information [file 41746_2020_294_MOESM1_ESM.pdf]

Supplementary Material

**Content Type**	**Title**
Certificate Course	*Computing for Medicine*
Curricular Lecture	*Intro to Machine Learning in Healthcare*
Curricular Lecture	*AI, Medicine, and the Future of Doctoring*
Student-Organized Workshop	*Design Thinking*
Student-Organized Lecture	*AI + Medicine — Mythology, Hype, and Reality*
Student-Organized Lecture	*Explainable AI in Healthcare: Interpretability, Humans-in-the-Loop, and policies and politics*
Student-Organized Lecture	*Doing No Harm: Ensuring AI Embodies Medical Ethics*
Student-Organized Lecture	*AI and the Future of Medicine*
Student-Organized Lecture	*Using Big Data to Measure and Improve Health Care*
Student-Organized Lecture	*Optimizing Cancer Care Using Artificial Intelligence (Cancelled due to COVID-19)*
Student-Organized Lecture	*Natural Language Processing in Clinical Systems*
Student-Organized Lecture	*UHN's Tech Stack 2.0*
Student-Organized Lecture	*Will Machine Learning and Big Data Solve Neuroscience's Problems?*
Student-Organized Lecture	*AI in Medicine: What Is and What Is To Come*
Student-Organized Lecture	*Machine Learning in Medicine and What Does it Mean for the Future?*

Supplementary Table 1: AI Content in the University of Toronto MD Program, 2018 to 2020
